# Using Accelerometer and GPS Data for Real-Life Physical Activity Type Detection

**DOI:** 10.3390/s20030588

**Published:** 2020-01-21

**Authors:** Hoda Allahbakhshi, Lindsey Conrow, Babak Naimi, Robert Weibel

**Affiliations:** 1 Department of Geography, Geographic Information Systems Unit, University of Zurich (UZH), Winterthurerstrasse 190, 8057 Zurich, Switzerland; Lindsey.Conrow@geo.uzh.ch (L.C.); Robert.Weibel@geo.uzh.ch (R.W.); 2 University Research Priority Program “Dynamics of Healthy Aging”, University of Zurich, Andreasstrasse 15, 8050 Zurich, Switzerland; 3 Department of Geosciences and Geography, University of Helsinki, P.O. Box 64, 00014 Helsinki, Finland; Naimi.b@gmail.com

**Keywords:** physical activity type, real-life, GPS, GIS

## Abstract

This paper aims to examine the role of global positioning system (GPS) sensor data in real-life physical activity (PA) type detection. Thirty-three young participants wore devices including GPS and accelerometer sensors on five body positions and performed daily PAs in two protocols, namely semi-structured and real-life. One general random forest (RF) model integrating data from all sensors and five individual RF models using data from each sensor position were trained using semi-structured (Scenario 1) and combined (semi-structured + real-life) data (Scenario 2). The results showed that in general, adding GPS features (speed and elevation difference) to accelerometer data improves classification performance particularly for detecting non-level and level walking. Assessing the transferability of the models on real-life data showed that models from Scenario 2 are strongly transferable, particularly when adding GPS data to the training data. Comparing individual models indicated that knee-models provide comparable classification performance (above 80%) to general models in both scenarios. In conclusion, adding GPS data improves real-life PA type classification performance if combined data are used for training the model. Moreover, the knee-model provides the minimal device configuration with reliable accuracy for detecting real-life PA types.

## 1. Introduction

In today’s societies, the increase in sedentary lifestyles in people’s homes and workplaces has caused severe health problems such as obesity and chronic diseases [1,2]. A physically active lifestyle can contribute to maintaining quality of life and preventing challenges related to people’s health status, particularly for older adults. Many studies have been designed to objectively measure physical activity (PA) using wearable sensors; however, they have been conducted in controlled conditions. The data collected under controlled conditions are unable to reproduce PA behavior as it happens in real-life [3]. Studying such behaviors in natural daily settings is therefore important in order to discover how daily PA types can affect health status.

Accurate PA type detection is a prerequisite to recognize humans’ daily activity behavior. Once we detect PA type, we can also estimate the other PA measures such as activity duration or level [4]. Detecting PA type helps to understand how much each activity type (e.g., walking or sitting) contributes to human physical and mental health. This also provides useful guidance regarding the amount of time that people should spend on a specific activity type to maintain their health. Moreover, PA type is a more understandable concept than PA level, particularly for laypersons [5]. Thus, it is imperative to improve daily PA type detection to identify humans’ daily PA patterns and their association with health outcomes.

During the past decade, rapid progress in wearable sensor technologies has facilitated long-term PA behavior monitoring in real-life conditions. Among the existing wearable sensors, three-dimensional (3D) accelerometers have gained the most attention. A 3D accelerometer (ACC) measures acceleration forces in y, x and z dimensions, and therefore can sense the status of a body’s motion or postures. Although the 3D accelerometer is the most common and informative sensor for PA type detection, it is challenging to accurately detect real-life activity types using only a single 3D accelerometer [5,6,7]. Researchers have extensively examined the usefulness of complementing accelerometer-based PA measures with additional sensors such as gyroscope, magnetometer, barometer and heart rate [8,9,10,11] or using multiple accelerometer devices on different body locations to improve the activity recognition [5,12]. However, these solutions entail mounting more devices on a person’s body or rendering data analysis more complex due to dealing with different sensors featuring different data formats and sampling rates. Moreover, few studies have investigated the role of global positioning system (GPS) data in informing classifiers for detecting PA types [5,13], despite the great potential that a GPS sensor might have in contributing spatial context information that could further facilitate the PA type detection process.

Combining GPS and accelerometer sensors has been useful in improving movement monitoring of humans, particularly in daily life. In the transport mode detection domain, the combination of GPS and accelerometer sensors is more useful than using each sensor individually, specifically in differentiating transport-related activities such as walking, cycling and running. In the PA literature, we can categorize the use of GPS sensors into two broad applications. The first application mainly focuses on utilizing GPS spatial coordinates to link PA behavior derived from accelerometer data to the location and relevant spatial data such as land use, walkability, green spaces, neighborhood and exposure in a geographic information systems (GIS) environment [14,15,16]. These links enhance our contextual knowledge of the relationship between objectively measured PA and physical and social environments [17,18,19,20,21]. The second application uses features such as time, distance, altitude and speed derived from GPS data to inform classifiers in PA detection [5,22,23,24,25]. However, few studies in the PA domain attempted to assess the potential benefit of using GPS data as additional input to PA type detection.

Previous studies indicated that utilizing GPS devices is a practical method to accurately estimate humans’ locomotion speed [26,27,28,29,30]. While adding GPS data (i.e., speed) to accelerometer data increases transport mode detection performance when differentiating between active and passive modes of transport [24,31,32,33], these studies rarely included different types of walking or cycling activities or different sub-types of the stationary class such as sitting, standing and lying. Although studies have included GPS speed to improve PA type detection for more fine-grained activities [5,13,34], they have a number of limitations that still have to be addressed.

Many of the models in the literature used data collected in controlled environments [5,13] to detect a limited number of activities from a small sample size [5,13,24,31,32,34]. Using GPS speed in combination with accelerometer data, models reliably detected activities that generate distinct accelerometer and GPS data profiles. However the models were unable to accurately detect activities with similar movement data profiles, such as non-level and level walking, which require different energy expenditure (EE) and have differing health impacts [6]. Exploiting GPS data to provide distinctive features would allow these similar types of activities to be distinguished. The previous studies have reported that the combination of GPS and accelerometer sensors generates better results for activity detection than using an accelerometer alone, but they did not fully discuss the role of the individual sensors in detail [5,24,32,34]. For example, it is unclear that to what extent adding GPS data improves activity recognition when using data collected in different environments (controlled and uncontrolled) or when using data from different sensor positions. It is also unknown whether adding GPS data addresses concerns about participant burden (e.g., wearing multiple sensors) during real-life data collection. To our knowledge, no study has explored the potential benefit of also using GPS spatial coordinates to classify PA type. The potential for combining GPS and accelerometer data to enhance real-life activity recognition is therefore a research area that is yet to be explored in detail.

This paper contributes to the body of literature on sensors and PA type detection first by calculating an informative elevation difference feature by linking the GPS spatial coordinates to GIS data, namely a digital elevation model (DEM), rather than using GPS speed alone. Second, we investigate the extent to which GPS sensors, in conjunction with accelerometer data, can enhance the prediction performance of detecting the major posture and transport-related motion activity types (sitting, standing, lying, walking, non-level walking, running and cycling). We then explore whether GPS data informs PA monitoring such that their inclusion minimizes the number of accelerometer devices that are required to reliably differentiate between the above posture and motion activity types under real-life conditions, with the aim of reducing participant burden. Finally, we advance research on real-life PA type detection through not only developing a single classification model, but also by assessing the contribution of GPS data in addressing the limitations of accelerometer sensor data and by studying the contribution of these sensors in detail within different realistic and stringent validation scenarios.

Our results provide insights that can assist future PA study design, especially when PA type detection is a focus. In particular, this research gives guidance regarding relevant data sources (accelerometer, GPS) and their usage, appropriate evaluation methods and optimal sensor positions for studies aiming to detect the major posture and transport-related motion activities.

## 2. Materials and Methods

### 2.1. Experimental Overview

The target PAs are lying, sitting, standing and walking on level ground at different speeds (slow, normal and fast), running, cycling, walking uphill, walking downhill, walking downstairs and walking upstairs. The rationale for selecting these target activities is to consider a subset of PAs from prior research including, (1) simple PAs classified by [35]. (2) Mobility-related activities of the International Classification of Functioning, Disability and Health (ICF) and (3) global body motion activities classified by [12]. (4) Activities that are commonly performed in everyday life and (5) activities that can cover different levels/intensities of PA and EE.

We used two study designs for data collection, semi-structured and real-life, to assess the transferability of the model trained with a semi-controlled data set on data collected in real-life conditions.

#### 2.1.1. Semi-Structured Protocol

**Figure 1:**
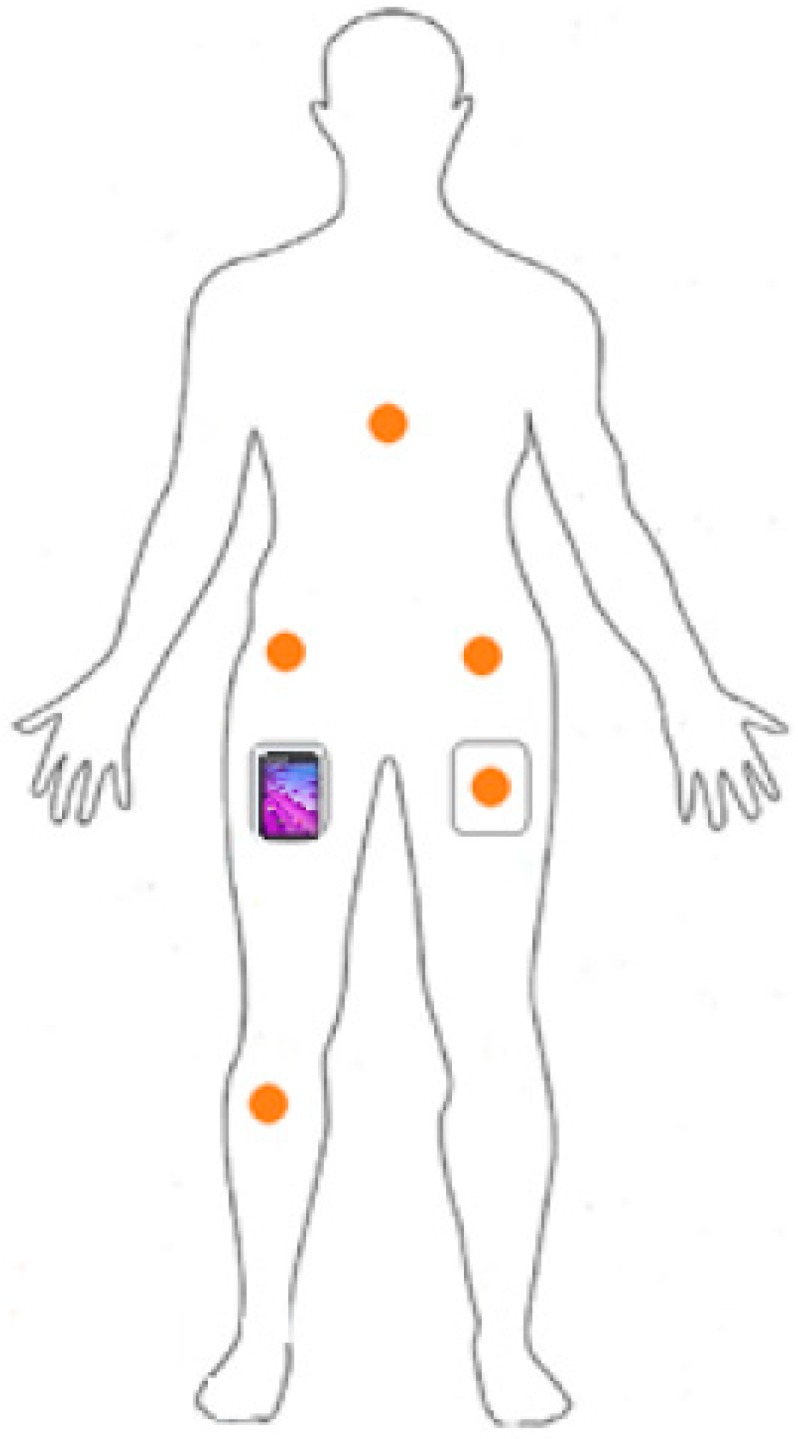
The location of smartphone and uTrail devices (orange circles) on the participants’ body.

Participants reported to the sport center of the University. After completing a questionnaire regarding their socio-demographic information and typical PA based on the Global Physical Activity Questionnaire (GPAQ) [36], they put the six devices on in the following configuration: one smartphone (Motorola Moto E, 2nd gen) inside their right pocket and five wearable customized uTrail devices [37] on different body locations including left and right hips, inside their left pocket, chest and right knee (Figure 1). Two elastic straps, each holding the uTrail, were adjusted around their chest and below their right knee. For the hip positions, we fixed the uTrail devices to their waistband using the device clip.

The uTrail device includes an audio sensor, a GPS sensor (uBlox UC530M) and an accelerometer that includes three magnetic field channels and three acceleration channels (ST Microelectronics LSM303D). The GPS recorded data at 1 Hz and has the ability of concurrent reception of up to three global navigation satellite systems (out of GPS (GPS = USA), Galileo (Galileo = European), GLONASS (GLONASS = Russia) and BeiDou (BeiDou = China)). The sampling rate for the accelerometer was 50 Hz. The uTrail device can be connected to a computer via a micro-USB port to download stored data; we were able to configure the device and retrieve the data via software developed for the uTrail. The smartphone and audio sensor data were not used in the present study. For all sensor positions except the right hip, the devices were oriented to have the y, x and z axes, recording acceleration data in the vertical, medio-lateral and antero-posterior direction of the body, respectively. For the right hip, the device was oriented to have the y, x and z axes, recording acceleration data in the vertical, antero-posterior and medio-lateral direction of the body, respectively.

Participants performed a number of activities, each completed twice in an outdoor area (see Appendix A, Table A1). They performed the motion activities at their own comfortable speed and were not restricted in this sense. We applied a direct observation approach for activity annotation using the “aTimeLogger” free app installed on a smartphone.

#### 2.1.2. Real-Life Protocol

The real-life experiment was conducted a few days after the participants completed the semi-structured protocol. Participants wore the devices in the same configuration as the semi-structured protocol and they were instructed to use the “aTimeLogger” app to make their own data annotation during the real-life data collection. No instruction regarding how to perform the activities was given to the participants. They performed the target activities in an outdoor environment as part of their daily life spontaneously and in a random order. The only criteria were to meet the required minimum time duration for each activity task described in (see Appendix A, Table A2) and perform the transport-related activities such as walking, cycling and jogging in two different environments, namely an urban area and a leisure area; this data collection protocol took 3 h on average. The total amount of labeled data collected in both protocols (semi-structured + real-life) is about 161 h (29,017,465 data recordings), corresponding to an average of 4.8 h labeled data for each participant (Table 1). We anonymized all data (with the personal data stored separately from the ACC and GPS data) and instructed the participants to perform all PAs away from their home and workplace, such that their home and workplace location could not be inferred from the GPS data. This dataset is not yet publicly available as we intend to use it in a future publication [38].

#### 2.1.3. Participants

**Figure A1:**
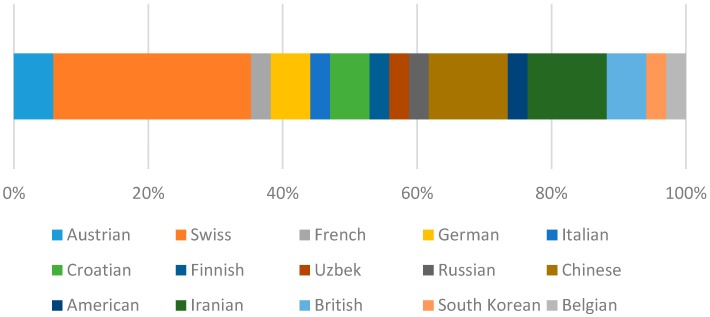
Nationality of the participants involved in the study.

A sample of 33 (20 male and 13 female) young participants ranging in age from 20 to 35 from 15 different countries (see Appendix A, Figure A1) participated in data collection (Table 2). As inclusion criteria, participants were required to be physically healthy and be able to walk and run without walking aids (self-report), and accept the instructions of the study protocol. The study was carried out following the rules of the Declaration of Helsinki of 1975. According to the rules of the University of Zurich (UZH) Ethics Policy, which are in accordance with the Swiss Human Research Act, it was not necessary to obtain separate ethics approval from the UZH Ethics Committee and our study was conducted in compliance with the ethical guidelines of the Philosophical Faculty of the University of Zurich. All participants provided written informed consent.

### 2.2. Model Development

#### 2.2.1. Accelerometer Preprocessing

After removing duplicates and missing values, we synchronized the data from five accelerometers. The synchronization was based on a sudden jump (i.e., “standing still-jump-standing still”) as introduced in [7] and was performed by the participant before and after performing each activity task, as instructed. The jump activity generated a distinguishable acceleration profile (i.e., peaks) within the standing still segments. We detected the peak acceleration of the start and end jumps, and aligned the data recordings of the five sensors based on those peaks. We used the start and end timestamps recorded by the “aTimeLogger” app to annotate the data. For each activity task, we removed 10 s before and after the activity segment to exclude data recorded during the sudden jump period for each activity. We also removed long stops (more than 1 s) within the motion activities. To do this, we firstly developed a threshold-based stop-move detection algorithm based on accelerometer data, secondly we found the stop segments longer than 1 s, thirdly we removed them from each motion activity segment and finally, we assigned the corresponding label to the raw accelerometer data of that segment. Visual inspection helped to ensure signal alignment to the corresponding activities.

We used an overlapping fixed size windowing technique to segment the labeled data. We applied a sensitivity analysis (i.e., we altered and tested different segment sizes) using segments of 2, 5, 10, 20, 30 and 60 s to investigate how robust the model’s classification performance was to the segment size. After signal segmentation, we calculated time and frequency domain features from each segment to use as inputs to the classifier. Time domain features are typically mathematical or statistical measures derived directly from the sensor data. To derive frequency domain features, the segment of sensor data must first be transformed into the frequency domain, normally using a fast Fourier transform (FFT). In total, we extracted 85 features from each sensor’s accelerometer data. The initial target features from accelerometer data include:

- Time domain features: mean, standard deviation and range of three axes and total acceleration, correlation among three axes, kurtosis, skewness and average absolute difference of three axes, number of observations falling within each of 10 bins of the three axes, time interval between local peaks and number of peaks of three axes.
- Frequency domain features using FFT: power spectral density, energy of the signal, mean of the first three dominant frequencies, amplitude of the first three dominant frequencies of three axes and total acceleration.

#### 2.2.2. GPS Preprocessing

The GPS data include latitude, longitude, date, time, horizontal dilution of precision (HDOP), vertical dilution of precision (VDOP), number of satellites, altitude and instantaneous speed. To preprocess the GPS data, we firstly removed duplicates and missing values. We used linear interpolation based on latitude, longitude and timestamps to fill the data gaps greater than 1 s between consecutive GPS fixes. We extracted an elevation value for each interpolated GPS point from a DEM to fill in the altitude value for the interpolated GPS points. A DEM is a representation of the altitude of the earth’s surface, today typically generated using remote sensing techniques such as stereo photogrammetry or laser scanning. We used the swissALTI3D DEM, which has a spatial resolution of 2 m and is provided by the Swiss national mapping agency swisstopo.

**Figure 2:**
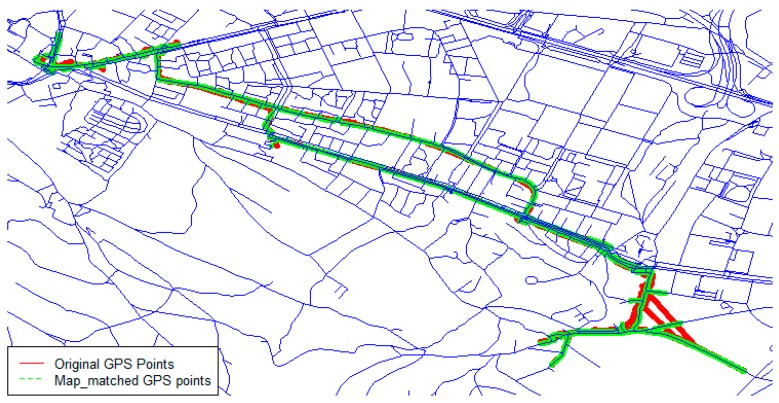
Map-matched global positioning system (GPS) points of data collected by a single participant in real-life using OpenStreetMap (OSM) data.

After filling gaps in the GPS data, it was important to keep the spatial error of GPS coordinates at a minimum. Map matching is a helpful solution to improve the spatial accuracy [39]. We used the point-to-curve geometric map-matching approach according to Quddus et al.’s (2007) categorization [39]. We applied an existing map matching algorithm on interpolated GPS data using road data obtained from OpenStreetMap (OSM) [40] and R software [41] (Figure 2).

Afterward, we used the map-matched GPS coordinates to derive an elevation value from swissALTI3D for each GPS point. SwissALTI3D is an accurate DEM, which describes the surface of Switzerland without vegetation and development and is updated every six years. We used ArcGIS software v.10.6.1 and the tool “Extract value to points” to assign an elevation value to each GPS point. We then used a weighted average filter to remove noise and outliers and smooth the extracted elevation data from DEM. We matched the GPS timestamps with the start and end timestamps of accelerometer segments to combine the GPS data with the accelerometer data. Finally, we calculated the average speed and elevation difference for each segment and appended these two GPS features to the accelerometer feature space.

#### 2.2.3. RF Model Development

We built two different training datasets, one using data from the semi-structured protocol only and another one using the combined dataset of both the semi-structured and real-life protocols, and used the random forest (RF) classifier to build the classification models in different scenarios (Table 3). For each scenario, we examined both single (accelerometer data only) and multi-sensor (accelerometer and GPS data) approaches to build the RF classification models. We built a general model that was trained with data obtained from all five sensor positions (chest, left hip, right hip, left pocket and right knee) and also five individual models, each trained with data from a single sensor position. Each accelerometer-based individual model used 85 features (see Section 2.2.1) for classification, and each accelerometer-based general model integrated features from all five sensors and used a total of 425 (85 × 5) features.

We grouped the activities of each protocol and detected seven classes including walking, non-level walking, running, cycling, sitting, standing and lying. We also validated the results using three approaches: Leave-One-Subject-Out (L1SO), k-fold cross validation and L1SO validation with the real-life data set. We tested different segment sizes (2, 5, 10, 20, 30 and 60 s) for general models to assess the effect of segment size on classification performance. The data analysis tasks were implemented using the R statistical computing software [41].

To report the classification performance, we used four metrics including accuracy, recall, precision and F1 (Equations (1)–(4)).

Accuracy = (True positive + True negative)/(True positive + True negative + False positive + False negative).(1)

Precision = True positive/(True positive + False positive).(2)

Recall = True positive /(True positive + False negative).(3)

F1 = 2 × precision × recall/((beta^2^ × precision) + recall). (4)

## 3. Results

**Figure 3:**
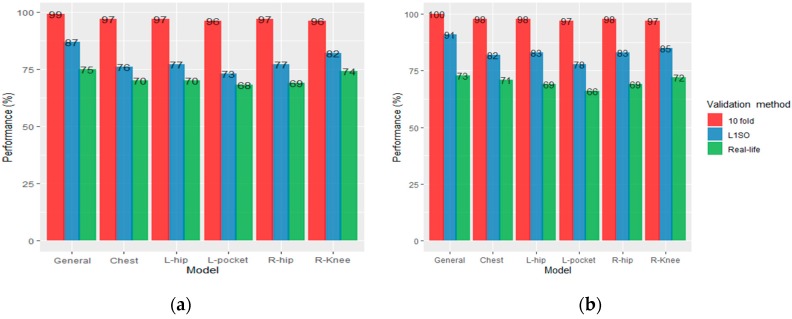
Overall accuracy of the RF classification models trained with semi-structured data, (**a**) accelerometer data only and (**b**) accelerometer and GPS data.

We presented the overall accuracies of the RF models (general model and individual models) as evaluated using L1SO, 10-fold cross validation and validation with a real-life dataset in Figure 3 and Figure 5. Based on the results, we realized that the L1SO cross validation (with a training or real-life dataset) led to more realistic results compared to 10-fold cross validation. The 10-fold cross validation always had the best performance (above 95%) for all models regardless of the sensor positions, training or testing dataset and there was significant difference between the classification accuracy measured using L1SO (with training or real-life dataset) and 10-fold cross validation. In other words, 10-fold cross validation produced artificially high scores for all models, therefore we focus on the results obtained by L1SO cross validation only.

### 3.1. Results for Scenario 1

Using L1SO cross validation (with training data) and accelerometer data only, the general model with 87% accuracy performed better than individual models. Among individual models, the knee position scored highest with 82% accuracy followed by left/right hip (77%), chest (76%) and left pocket (73%). We observed a dramatic drop in accuracy under real-life dataset when using L1SO cross validation, indicating that the model trained with semi-structured data could weakly predict PA types in real-life (Figure 3a).

Adding GPS data to the accelerometer data improved the classification performance for all models validated by L1SO of the training dataset. The overall accuracy of hips and chest, pocket, general and knee positions increased by 6%, 5%, 4% and 3%, respectively. However, similar to accelerometer-based models, the classification performance decreased for all models when testing on real life data. General model performed the best with 73% accuracy followed by knee (72%), chest (71%), left/right hips (69%) and pocket (66%; Figure 3b).

**Figure 4:**
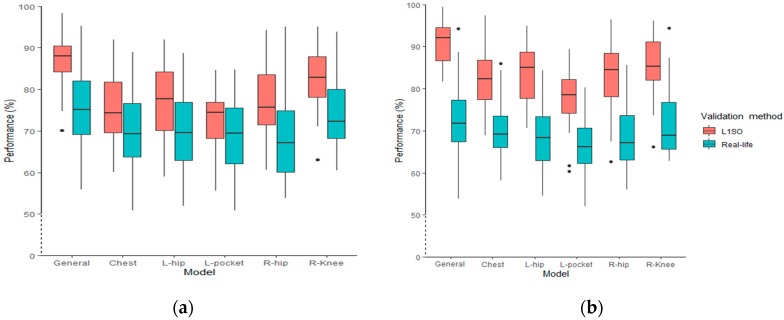
The distribution of overall accuracy among all participants for the RF classification models trained with semi-structured data, (**a**) accelerometer data only and (**b**) accelerometer and GPS data.

Using L1SO of the training dataset, the overall accuracy for the general models ranged from 70% to 98% (using accelerometer data only) and from 81% to 99% (using accelerometer data combined with GPS data). Testing the general models with real-life data, the classification performance was between 56% and 95% and 56% and 95% using accelerometer data and ACC + GPS data, respectively. The interquartile range (IQR) of L1SO and the related real-life validation partially overlapped for all models excluding the general model when using accelerometer data only (Figure 4a). Conversely, there was no overlap between the IQR of L1SO and its related real-life validation when we added GPS data (Figure 4b). In addition, using multi-sensor data (Figure 4b) generated more outliers compared to using accelerometer data only (Figure 4a). The distribution range of the general and individual position models does not show a significant difference between Figure 4a,b. Results show that in an ideal situation (i.e., fewer GPS gaps and complete OSM data), adding GPS data could increase the overall classification accuracy for L1SO of training dataset by 15%.

The general RF model using accelerometer data only detected lying, sitting, standing and running with high recall, precision and F1. However, the model obtained the lowest performance for non-level walking followed by walking and cycling (highlighted in bold in Table 4). Adding features derived from GPS data (speed and elevation differences) to the accelerometer feature space significantly improved the recall, precision and F1 for non-level walking, walking and cycling (highlighted in bold in Table 5).

#### Feature Importance

**Figure A2:**
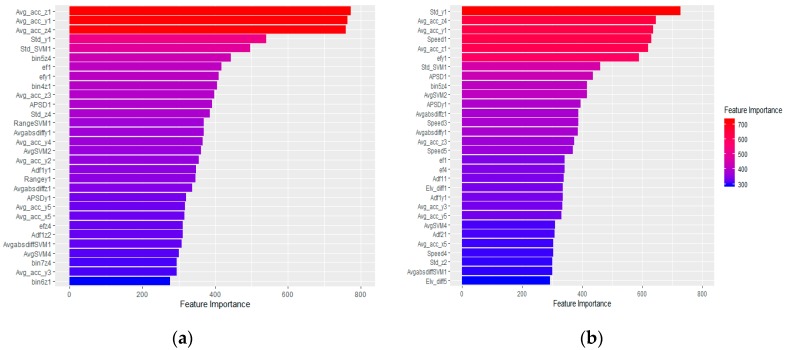
Top 30 important features of a participant’s general RF model (with the highest GPS contribution) trained with semi-structured data (see the feature description in Table A3). (**a**) Accelerometer data only and (**b**) accelerometer and GPS data.

Using accelerometer data only, the mean acceleration along the vertical and medio-lateral axes, standard deviation and energy of the signal of total acceleration and of the vertical axis, the number of observations falling within the fourth bin of the medio-lateral axis from the chest sensor’s data; the mean acceleration along the medio-lateral axes of the left hip and pocket sensor’s data and the number of observations falling within the fifth bin of the medio-lateral axis from the pocket’s data were the top 10 best features for the general RF model (see Appendix B, Figure A2a).

Though the order of important features varied according to the different individual models, the mean acceleration along the vertical and medio-lateral axes, as well as power spectral density, and energy and amplitude of the first dominant frequency of total acceleration fell within the top 10 features for all individual models. We also observed that mean acceleration along the antero-posterior axis and total acceleration, average absolute difference of total acceleration, standard deviation of total acceleration, vertical and medio-lateral axes, energy of the signal along the vertical and medio-lateral axes, amplitude of the second dominant frequency of total acceleration, number of observations falling within the fourth, fifth and seventh bin of the medio-lateral axis and range of acceleration along the medio-lateral axis are among the top 10 features among different individual models.

Using the accelerometer and GPS data, excluding the features derived from GPS data, a similar feature importance pattern was seen for the general (see Appendix B, Figure A2b) and individual models.

### 3.2. Results for Scenario 2

**Figure 5:**
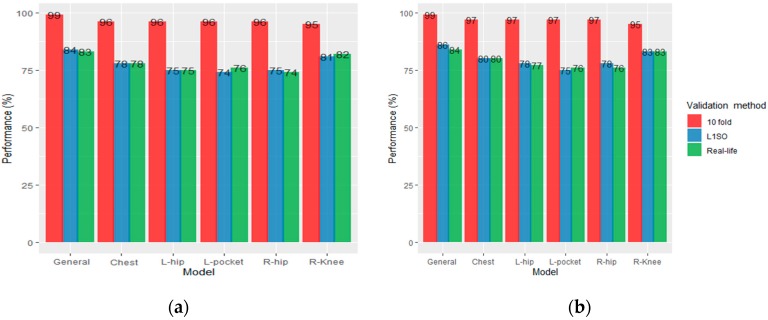
Overall accuracy of the RF classification models trained with combined dataset, (**a**) accelerometer data only and (**b**) accelerometer and GPS data.

In Scenario 2, for each participant, we combined all collected data in the semi-structured and real-life settings, and built the training data or “combined dataset” (Figure 5).

Using L1SO cross validation (with training data) and accelerometer data only, the general model achieved 84% accuracy, a 3% decrease compared to the result obtained by using semi-structured data in training. Among individual models, the knee position again scored highest with 81% accuracy followed by chest (78%), hips (75%) and left pocket (74%). Using the combined data for training the RF, compared to Scenario 1, the model performance for chest and pocket positions slightly increased by 2% and 1%; whereas, it decreased by 3% and 1% for hips and knee position, respectively (Figure 5a).

Adding GPS data to the accelerometer data improved the classification performance for all models validated by L1SO of the training dataset by 2% with the exceptions of 1% for the pocket model and 3% for the hips position. This scenario also performed better with the real-life data, and ACC + GPS resulted in stable classification performance, unlike Scenario 1 where the performance dramatically dropped for all models. The general, knee, chest, pocket and right hip, and left hip models achieved 84%, 83%, 80%, 76% and 77% overall accuracy, respectively (Figure 5b).

**Figure 6:**
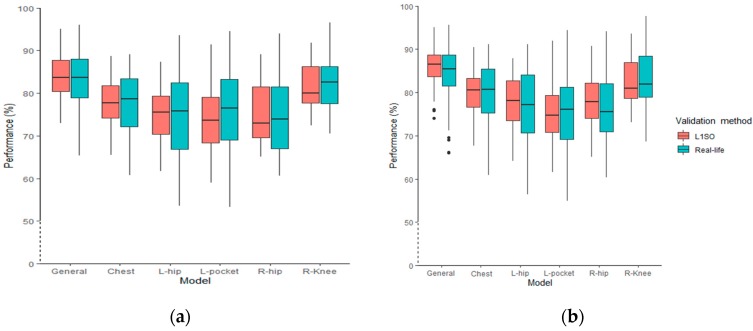
The distribution of overall accuracy among all participants for the RF classification models trained combined dataset, (**a**) accelerometer data only and (**b**) accelerometer and GPS data.

The boxplots for the models’ performance when using the combined dataset for training RF models shows that the overall accuracy ranges from 73% to 95% and from 74% to 95% when using accelerometer data only and when using data from both accelerometer and GPS sensors, respectively, and evaluated by L1SO (Figure 6). Testing the general models with real-life data, the overall accuracy ranges from 65% to 95% (Figure 6a) and 66% to 96% (Figure 6b). The IQR of L1SO and the related real-life validation overlapped for all models. The overall accuracies follow a similar distribution trend for both Figure 6a,b, regardless of sensor positions and validation methods. Both hip positions and the pocket models had the widest distribution followed by chest, knee and general models. Adding GPS data produced more outliers, as was the case in Scenario 1.

The confusion matrix for the participant with the highest GPS contribution (4%) shows that the most misclassification occurred for non-level walking and walking activities when using accelerometer data only and L1SO validation (highlighted in bold in Table 6). Similar to Scenario 1, adding GPS features improved the classification performance by reducing the misclassification errors for these two activities (highlighted in bold in Table 7).

#### Feature Importance

**Figure A3:**
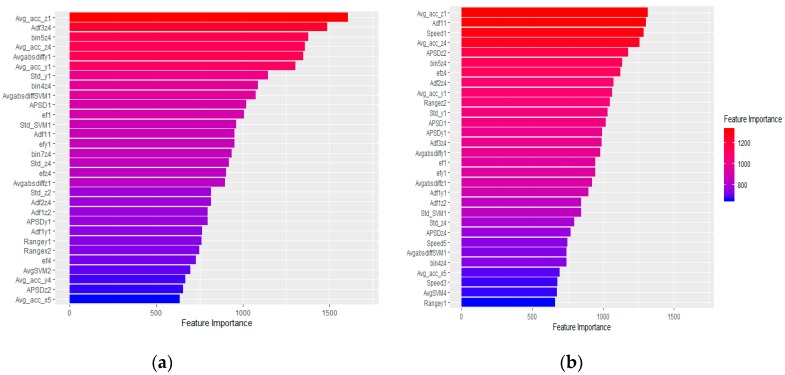
Top 30 important features of a participant’s general RF model (with the highest GPS contribution) trained with combined data (see the feature description in Table A3). (**a**) Accelerometer data only and (**b**) accelerometer and GPS data.

As in Scenario 1, mean acceleration along the vertical and medio-lateral axes, standard deviation of acceleration along the vertical axis from the chest sensor’s data, mean acceleration along the vertical axis and number of observations falling within the fifth bin of the medio-lateral axis from the pocket sensor’s data fell within the top 10 features for the general model when using accelerometer data only (see Appendix B, Figure A3a). The average absolute difference of total acceleration and acceleration along the vertical axis, power spectral density of total acceleration from the chest’s sensor data, number of observations falling within the fourth bin of the medio-lateral axis and amplitude of the third dominant frequency of acceleration along the medio-lateral axis from the pocket’s sensor data were also among the top 10 important features for accelerometer data. The individual models’ importance pattern for the top 10 features was similar to the individual models’ feature importance in Scenario 1. There was again variation in the order of feature importance depending on the different individual models. The mean acceleration along the medio-lateral axis and power spectral density of total acceleration were among the top 10 features of all individual models.

Using accelerometer and GPS data, excluding the features derived from GPS data, we observed a similar feature importance pattern for the general (see Appendix B, Figure A3b) and individual models.

### 3.3. Sensitivity Analysis on Segment Size

**Figure 7:**
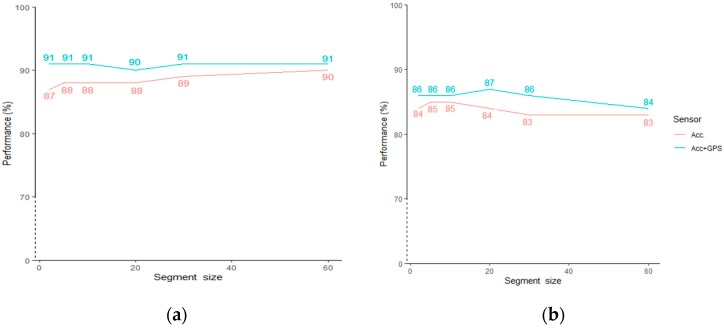
Sensitivity analysis on segment size, (**a**) general model trained with semi-structured dataset and (**b**) general model trained with combined dataset.

We performed sensitivity analysis on different segment sizes using L1SO of training data to determine how sensitive the models are to the segment size. For both scenarios, we tested segment sizes of 2, 5, 10, 20, 30 and 60 s and performed L1SO cross validation on the general RF models. The results show that performance starts to converge with larger window size in (a) whereas (b) has the widest gap at 20 and 30s. Overall, there are slight changes ranging from 1% to 3% for the models’ performance when using different segment sizes (Figure 7).

## 4. Discussion

### 4.1. Discussion of Results

The aim of this study was to investigate the extent to which using GPS sensor data, in conjunction with accelerometer data, enhances the prediction performance in detecting the major posture and transport-related motion activity types (sitting, standing, lying, level walking, non-level walking, running/jogging and cycling). Moreover, this study explored how adding GPS data allows the number of sensor devices to be minimized in PA monitoring.

The validation results show that using standard 10-fold cross validation, which allows data from the same participant in the test and training set produces artificially high accuracy scores. Though 10-fold cross validation is commonly used, it is a weak evaluation method, while L1SO cross validation corresponds to a more realistic setting in which the algorithm would be applied. In practical use, the data from a particular participant are never used as training data to classify another piece of data from that same participant, but will instead be used to classify data from another participant. Hence, it is likely that L1SO scored lower than 10-fold because different participants have different ways of performing individual activities. For these reasons, we recommend against using the 10-fold cross validation method in the PA type detection.

Adding GPS data to the general model improved the accuracy in Scenario 1, although the developed models showed a dramatic decrease when evaluated with L1SO applied on the real-life dataset. Performance decreased by 12% when using accelerometer only, and by 18% when using both accelerometer and GPS. This result indicates that adding GPS data to accelerometer data produces significant generalization error when tested with a real-life dataset. The generalization error may result from performing the activities in different real-life environments (leisure and urban), impacting the variable accuracy of GPS data. Urban areas in particular can affect GPS signal reception and therefore generate more GPS gaps and uncertainty in the data. Having more outliers in the boxplots when adding GPS data is also a manifestation of the increase in data uncertainty (Figure 4a). Moreover, incomplete OSM data is more often encountered in leisure (i.e., rural) environments, which influences the outcome of map matching GPS data collected in those areas. Thus, models developed using semi-structured data are weakly transferable to the data collected in real life, particularly when we add GPS data to the training data. The distribution of overall accuracy among all participants also shows the above-mentioned conclusion; by adding GPS data, there is a larger gap between IQR of L1SO and its related real-life validation for the general model (Figure 4b).

We used the combined dataset to train models in Scenario 2 to improve the transferability of our models for a real-life dataset and address the overfitting issue. In machine learning, overfitting refers to when a model learns the training data very well but performs weakly on a new dataset. Compared to the semi-structured dataset, there is more variation in the combined dataset, which explains the overall decrease in overall accuracy of the models between Scenarios 1 and 2. Using the combined data, the models showed comparable accuracy when evaluated by a L1SO of the training data and when evaluated with the real-life dataset. Testing the models on the real-life dataset of an unseen participant in the training data resulted in an overall accuracy of 83% for the accelerometer-based model (decreasing by only 1% compared to the result obtained by using L1SO validation of the training data) and 86% for the ACC + GPS based model. We therefore conclude that the new models trained with the combined dataset generate robust models with reproducible classification performance for real-life data from new subjects. The high degree of overlap between IQR of L1SO and the related real-life validation for all models in Scenario 2 also supports this conclusion. The advantage of using the combined dataset rather than the semi-structured dataset for training the model is that there is less generalization error in the classification performance when we use a real-life (i.e., a new) dataset for testing. This supports the results by Ermes et al. (2008) that in order to build a model that performs reliably on a real-life dataset, it is necessary to include labeled data collected in real-life in the training data [34]. It also explains why Scenario 2 performed better than Scenario 1 on the real-life data, with ACC + GPS increasing real-life performance.

Regarding the features, we did not apply any feature selection or dimensionality reduction algorithms as we used the random forest as a classifier, which performs feature selection throughout the classification process. Therefore, using the random forest classifier, the high number of features for general models does not lead to oscillations of the classification. We also used the R package ranger [42], which is a faster and more memory-efficient implementation of random forests, to improve the models’ processing time. In general, when excluding GPS features, similar time and frequency domain features from accelerometer data appeared in all models, though the importance changes based on the sensor’s position. The top 10 important features that gained the highest frequency among all models include the mean acceleration of the vertical, medio-lateral and antero-posterior axes; energy of acceleration along the vertical and medio-lateral axes; standard deviation of vertical axis and the average absolute difference, standard deviation, energy, power spectral density and amplitude of the first dominant frequency of total acceleration.

We performed a sensitivity analysis on six different segment sizes to assess the transferability of our models on data extracted from different time intervals. The highest GPS contribution to the classification performance was for the segment size of 2 s (4%) in Scenario 1 and for the segment sizes of 20 and 30 s (3%) in Scenario 2. Adding GPS data resulted in a high accuracy of 91% for all segment sizes except 20 s (90%) in Scenario 1. In Scenario 2, using multi-sensor data led to the highest accuracy of 87% when 20s segments were used. As there are only slight changes ranging from 1% to 3% for the models’ performance when using different segment sizes, we could conclude that our models were stable and robust to the segment size. Using the longest, 60 s segment size, the ACC + GPS models in Scenario 1 and 2 reached 91% and 84% overall accuracy, respectively. This demonstrated that our models would be useful when collecting data with storage and battery limited devices (such as smartphones), which have limitations in recording sensor data at high sampling rates during long-term PA monitoring.

Comparing the five individual models, each trained on data from a single sensor position, showed that hips and chest models generate comparable accuracy with and without adding GPS data. For both hip positions, we usually gained similar classification performance, although we asked participants to wear the hip devices in different orientations. This shows that the orientation does not have a significant influence on the overall classification performance when using hip positions. However, looking more in detail, we found that the two hip models have distinguishable performance for different participants in detecting different activities. For example, the left hip model detects sitting activity better than the right hip model for some participants. The pocket position usually performed worse than other positions, possibly because the device in this position was not fixed as participants simply put the device in their pocket, which could cause flipping or rotating the device during activity performance. The knee model performed best both when using accelerometer only and when using multi-sensor data in both scenarios. In Scenario 2, the knee model showed comparable performance with the general model and achieved an accuracy above 80%. It also gained the most similar IQR compared to its related general model in this scenario. Moreover, in an ideal situation, the knee multi-sensor model obtained an overall accuracy of 94% for detecting the major posture and motion activities (see Appendix B, Table A4) when evaluated by L1SO on training data. This indicates that adding GPS data to knee-positioned accelerometer data provides classification performance with high accuracy, which further suggests that participant burden might be reduced as the number of sensor devices can be minimized for PA type detection.

### 4.2. Contributions and Limitations

This study has several strengths and limitations worth noting. A general strength of this study is that we comprehensively investigated the contribution of adding GPS data to enhance accelerometer-based PA type classification, as discussed above. We accurately detected activities that help to discover humans’ daily activity behavior. For instance, Ermes et al., (2008) noted that the majority of data collected in the real-life environment by their participants (78%) include lying, sitting and standing, and emphasized the importance of detecting these three stationary activity types in real-life. However, they grouped sitting and standing to one group, as their model could not reliably distinguish these two activities [34]. There is a causal effect between spending too much time on these activities and the risk of negative health impacts such as diabetes or obesity. Detecting stationary activities, therefore, allows measurement of the amount of time people can spend on other more health-enhancing activities in their daily life. Related to this, Nguyen et al., (2013) were unable to accurately detect activities with similar GPS speed and accelerometer data profiles that require different EE and have a different health impact such as non-level and level walking [6]. In order to better detect these activities, in addition to GPS speed, we extracted another distinctive feature (elevation difference) by linking GPS spatial coordinates to DEM data.

Compared to most studies, which use a small sample size, we employed a large sample of thirty-three people that generated a comprehensive training dataset in terms of the diversity of the subjects’ physical characteristics and also inspires confidence in our results. We used a customized light portable device with embedded accelerometer and GPS sensors for data collection, which overcomes the drawbacks of using smartphones or multiple devices. Using smartphones reduces the burden on the participant [24,31], as there is no need to carry extra devices; however, smartphones’ limited battery and storage makes long-term activity monitoring problematic. Moreover, user interaction with the smartphone, such as making a phone call or sending a text message, can affect the sensor’s data quality. Applying multi-devices [5,13,32,34] also entails carrying more devices and therefore a great burden on participants, particularly in real-life PA monitoring. In real-life experiments, well-designed data collection logistics are necessary to ensure that the process is minimally invasive for participants, while providing suitable data quality for researchers. Studies have addressed the question of how different body locations of accelerometers’ can influence the performance of PA type detection [10,12,43]. However, it previously remained unknown how GPS sensor data can help in providing minimum a device configuration when used in combination with accelerometer data. Our examination of five device locations showed that the model developed using GPS and accelerometer data from a knee-worn device produces comparable high accuracy (above 80%) to the model developed by using data from multiple devices.

This study has some limitations that should be addressed in future research. The models that included GPS data are limited to detect outdoor activities because there are limitations regarding GPS signal reception in indoor environments and DEM data are only available for outdoor environments. We applied a high resolution DEM (2 m ground resolution) to extract elevation information for each GPS point; using a DEM with low resolution may not lead to similar results. We only used linear interpolation and point-to-curve geometric map matching to preprocess the GPS data. Other interpolation and map matching methods might help to advance the classification performance. The high performance of the developed models, however, can be achieved only when GPS data with few gaps and complete OSM data are available. Low data quality might lead to unreliable PA classification performance. Moreover, as in all such studies, the classification results depend on the target activities and study settings; selecting other activities and experimental conditions might lead to different outcomes. Though we accurately detected three major sub-types of postures (i.e., sitting, standing and lying), we did not aim to detect other sub-types of posture activity such as active standing (which occupies significant percentages of human daily activities) or complex activities [44]. In future studies, a wider range of activities should be included to provide more information about health-related daily PAs. Though we achieved a high classification performance using the RF classifier, applying other advanced machine learning models such as recurrent neural networks including long short-term memory (LSTM) networks [45,46] and comparing their performance may be considered as a future study. Finally, we trained the models using data collected by young healthy adults only. To what extent these models are transferable to older adults is a research question that we would like to answer in a future study.

## Figures and Tables

**Table 1:** Labeled data collected for the study.

Dataset	Total Acc. Data	Total GPS Data	Acc. Data per Person	GPS Data per Person
Semi-structured	61.6 h (11,098,581)	59.6 h (214,628)	1.8 h (336,320.6)	1.8 h (6503.879)
Real-life	99.5 h (17,918,884)	101.5 h (365,631)	3 h (542,996.5)	3 h (11,079.73)
Total	161 h (29,017,465)	161 h (580,259)	4.8 h (879,317.1)	4.8 h (17,583.61)

**Table 2:** Physical characteristics of the participants involved in the study.

Physical Characteristics	Mean (SD)
No. (F/M)	33 (13/20)
Age (year)	29 ± (5.6)
Height (cm)	173 ± (10.05)
Weight (kg)	67 ± (9.8)
BMI (kg·m^−2^)	22 ± (1.9)

**Table 3:** Scenarios for separating data into a train and test data set and the corresponding validation method.

Scenario No.	Training Dataset	Validation Method and Test Data
Scenario 1	Semi-structured dataset	L1SO cross validation on semi-structured data L1SO cross validation on real-life data k-fold cross validation on semi-structured data
Scenario 2	Combined semi-structured and real-life dataset	L1SO cross validation on combined data L1SO cross validation on real-life data k-fold cross validation on combined data

**Table 4:** Confusion matrix of a participant (with the highest GPS contribution) when using accelerometer data only (Scenario 1).

Accelerometer Only	Cycle	Lie	N_Walk	Run	Sit	Stand	Walk	Recall	Precision	F1
**Cycle**	168	0	9	0	0	0	0	**78**	**95**	**85**
**Lie**	0	124	0	0	0	1	0	99	99	99
**N_walk**	0	0	209	0	0	0	163	**42**	**56**	**48**
**Run**	1	0	0	113	0	0	0	100	99	100
**Sit**	0	1	0	0	108	0	0	99	99	99
**Stand**	0	0	0	0	1	62	0	98	98	98
**Walk**	47	0	279	0	0	0	394	**71**	**55**	**62**

**Table 5:** Confusion matrix of a participant (with the highest GPS contribution) when using accelerometer and GPS data (Scenario 1).

Accelerometer & GPS	Cycle	Lie	N_Walk	Run	Sit	Stand	Walk	Recall	Precision	F1
**Cycle**	165	0	10	0	0	0	0	**98**	**94**	**96**
**Lie**	0	124	0	0	0	1	0	99	99	99
**N_walk**	0	0	278	0	0	0	89	**58**	**76**	**66**
**Run**	1	0	0	112	0	0	0	100	99	100
**Sit**	0	1	0	0	107	0	0	100	99	100
**Stand**	0	0	0	0	0	66	0	99	100	99
**Walk**	2	0	192	0	0	0	523	**85**	**73**	**79**

**Table 6:** Confusion matrix of a participant (with the highest GPS contribution) when using accelerometer data only (Scenario 2).

Accelerometer only	Cycle	Lie	N_Walk	Run	Sit	Stand	Walk	Recall	Precision	F1
**Cycle**	743	0	2	0	0	0	0	100	100	100
**Lie**	0	185	1	0	1	0	0	99	99	99
**N_walk**	2	0	800	1	0	0	91	**77**	**89**	**83**
**Run**	0	0	0	320	0	0	0	99	100	100
**Sit**	0	1	0	0	170	1	0	99	99	99
**Stand**	0	1	0	0	0	157	0	99	99	99
**Walk**	0	0	233	2	0	1	885	**91**	**79**	**84**

**Table 7:** Confusion matrix of a participant (with the highest GPS contribution) when using accelerometer and GPS data (Scenario 2).

Accelerometer & GPS	Cycle	Lie	N_Walk	Run	Sit	Stand	Walk	Recall	Precision	F1
**Cycle**	738	0	0	0	0	0	0	100	100	100
**Lie**	0	186	1	0	0	0	0	99	99	99
**N_walk**	1	0	810	1	0	0	63	**89**	**93**	**91**
**Run**	0	0	0	318	0	0	1	99	100	99
**Sit**	0	1	0	0	166	1	0	98	99	99
**Stand**	0	1	0	0	3	158	0	99	98	98
**Walk**	1	0	97	2	0	1	1018	**94**	**91**	**93**

## Appendix A

**Table A1:** Activity tasks for the semi-structured data collection.

Activity Task (I)	Activity Task (II)	Duration: 16.5 + 16.5 + 33 1.5 h
First step: Walking at different speed		
Jump and stand still for 5 s at starting point	Jump and stand still for 5 s at starting point	3 + 3
Walk at SLOW speed	Walk at NORMAL speed	
Turn left at turning point (sharp turn)	Turn right at turning point (smooth turn)	
Walk at SLOW speed	Walk at NORMAL speed	
Stop at the stop point for 5 s	Stop at the stop point for 5 s	
Walk at FAST speed	Walk at FAST speed	
Stop at the stop point for 5 s	Stop at the stop point for 5 s	
Walk at NORMAL speed	Walk at SLOW speed	
turn left at point (smooth turn)	turn right at turning point (sharp turn)	
Walk at NORMAL speed	Walk at SLOW speed	
Stand still for 5 s at end point and jump	Stand still for 5 s at starting point and jump	
Second step: Running		
Jump and stand still for 5 s at starting point	Jump and stand still for 5 s at starting point	1.5 + 1.5
Run at self-paced speed	Run at self-paced speed	
Turn left at turning point (sharp turn)	Turn right at turning point (smooth turn)	
Run at self-paced speed	Run at self-paced speed	
Stop at the stop point for 5 s	Stop at the stop point for 5 s	
Run at self-paced speed	Run at self-paced speed	
Turn left at turning point (smooth turn)	Turn right at turning point (sharp turn)	
Run at self-paced speed	Run at self-paced speed	
Stand still for 5 s at starting point and jump	Stand still for 5 s at starting point and jump	
Third step: Cycling		
Jump and stand still for 5 s at starting point	Jump and stand still for 5 s at starting point	1.5 + 1.5
Get on the cycle	Get on the cycle	
Cycle at self-paced speed	Cycle at self-paced speed	
Turn left at the turning point	Turn right at the turning point	
Cycle at self-paced speed	Cycle at self-paced speed	
Turn left at the turning point	Turn right at the turning point	
Stop at the ending point	Stop at the ending point	
Get off the cycle	Get off the cycle	
Stand still for 5 s at starting point and jump	Stand still for 5 s at starting point and jump	
Fourth step: Stairs walking		
Jump and stand still for 5 s at starting point	Jump and stand still for 5 s at starting point	1 + 1
Walk upstairs at normal speed	Walk upstairs at normal speed	
Stand still for 5 s after first floor	Stand still for 5 s after first floor	
Walk upstairs at normal speed	Walk upstairs at normal speed	
Stand still for 5 s at ending point and jump	Stand still for 5 s at ending point and jump	
Jump and stand still for 5 s at starting point	Jump and stand still for 5 s at starting point	1 + 1
Walk downstairs at normal speed	Walk downstairs at normal speed	
Stand still for 5 s after first floor	Stand still for 5 s after first floor	
Walk downstairs at normal speed	Walk downstairs at normal speed	
Stand still for 5 s at ending point and jump	Stand still for 5 s at ending point and jump	
Fifth step: Walking at different slopes		
Jump and stand still for 5 s at starting point	Jump and stand still for 5 s at starting point	2 + 2
Walk uphill at normal speed	Walk uphill at normal speed	
Stand still for 5 s at ending point and jump	Stand still for 5 s at ending point and jump	
Jump and stand still for 5 s at starting point	Jump and stand still for 5 s at starting point	2 + 2
Walk downhill at normal speed	Walk downhill at normal speed	
Stand still for 5 s at ending point and jump	Stand still for 5 s at ending point and jump	
Sixth step: Sedentary activities		
Sit		
Jump	Jump	1 + 1
Go from standing position to sitting	Go from standing position to sitting	
Sit for 1 min	Sit for 1 min	
Go from sitting position to standing	Go from sitting position to standing	
Stand	Stand	
Jump	Jump	
Stand		
Jump	Jump	1 + 1
Stand for 1 min	Stand for 1 min	
Jump	Jump	
Lie		
Jump	Jump	1 + 1
Go from standing position to sitting	Go from standing position to sitting	
Sit	Sit	
Go from sitting position to lying on your back	Go from sitting position to lying on your back	
Lie on your back for 1 min	Lie on your back for 1 min	
Go from lying on your back to sitting position	Go from lying on your back to sitting position	
Sit	Sit	
Go from sitting position to standing	Go from sitting position to standing	
Stand	Stand	
Jump	Jump	

**Table A2:** Activity tasks for the real-life data collection.

Activity	Minimum Duration (Minute)	Location
Sedentary activities		
Lying	1	Outdoors (e.g., on a bench)
Sitting	1	Outdoors (not in a vehicle)
Standing	1	Outdoors (not in a vehicle)
Non-level walking		
Walking uphill	2	Outdoors
Walking downhill	2	Outdoors
Walking downstairs	2 floors (8 steps each)	Outdoors
Walking upstairs	2 floors (8 steps each)	Outdoors
Transport-related activities		
Walking, level ground	5	Leisure area (e.g., park)
	5	Urban area (e.g., street sidewalk)
Cycling, level ground	5	Leisure area (e.g., park)
	5	Urban area (e.g., street bike path)
Running, level ground	1	Leisure area (e.g., park)
	1	Urban area (e.g., street sidewalk)

## Appendix B

**Table A3:** List of features appearing in Figure A2 and Figure A3.

Feature Notation	Description
1, 2, 3, 4, 5	1 = Chest sensor’s data 2 = Knee sensor’s data 3 = left hip sensor’s data 4 = left pocket sensor’ data 5 = right hip sensors’ data (e.g., ef1 = energy of total acceleration derived from chest sensor’s data)
SVM	Total acceleration
Avg-SVM, Avg-acc-x, Avg-acc-y, Avg-acc-z	Mean of total acceleration and each axis
Std-SVM, Std-x, Std-y, Std-z	Standard deviation of total acceleration and each axis
BinN, BinNx, BinNy, BinNz	Number of observations falling within the Nth bin of total acceleration and each axis
Avgabsdiff, Avgabsdiffx, Avgabsdiffy, Avgabsdiffz	Average absolute difference of total acceleration and each axis
RangeSVM, Rangex, Rangey, Rangez	Range of total acceleration and each axis
APSD, APSDx, APSDy, APSDz	Power spectral density of total acceleration and each axis
ef, efx, efy, efz	Energy of total acceleration and each axis
ADF1, ADF1x, ADF1y, ADF1z	Amplitude of the first dominant frequency of total acceleration and each axis
ADF2, ADF2x, ADF2y, ADF2z	Amplitude of the second dominant frequency of total acceleration and each axis
ADF2, ADF2x, ADF2y, ADF2z	Amplitude of the third dominant frequency of total acceleration and each axis

**Table A4:** Confusion matrix of a participant when using accelerometer and GPS data for knee position. (Scenario 2).

Accelerometer & GPS	Cycle	Lie	N_Walk	Run	Sit	Stand	Walk	Recall	Precision	F1
**Cycle**	1245	0	8	1	0	0	11	98	98	98
**Lie**	0	196	0	0	0	0	0	100	100	100
**N_walk**	21	0	536	0	0	1	145	76	91	83
**Run**	0	0	3	254	0	0	3	98	99	98
**Sit**	1	0	1	0	195	2	0	98	93	95
**Stand**	0	0	0	0	15	259	0	95	99	97
**Walk**	0	0	43	1	0	0	1029	96	87	91
